# The Linoleic Acid Content of the Stratum Corneum of Ichthyotic Golden Retriever Dogs Is Reduced as Compared to Healthy Dogs and a Significant Part Is Oxidized in Both Free and Esterified Forms

**DOI:** 10.3390/metabo11120803

**Published:** 2021-11-26

**Authors:** Iuliana Popa, Audrey Solgadi, Didier Pin, Adrian L. Watson, Marek Haftek, Jacques Portoukalian

**Affiliations:** 1 Analytic and Biological Lipid Systems (Lip(Sys)2), Faculty of Pharmacy, University Paris-Saclay, 92296 Chatenay-Malabry, France; 2 Technical Platform-Therapeutical Innovation Institute (IPSIT)-UMS 3679 CNRS, Faculty of Pharmacy, University Paris-Saclay, 92290 Chatenay-Malabry, France; audrey.solgadi@universite-paris-saclay.fr; 3 Interactions Cells Environment, UPSP 2016.A104, VetAgro Sup Veterinary Campus, University of Lyon, 69280 Marcy l’Etoile, France; didier.pin@vetagro-sup.fr; 4 Royal Canin SAS, 30470 Aimargues, France; adrian.watson@effem.com; 5 Laboratory of Tissue Biology and Therapeutic Engineering(LBTI), CNRS UMR 5305, University of Lyon 1, 69367 Lyon, France; marek.haftek@univ-lyon1.fr; 6 Faculty of Medicine, University of Lyon 1, 69622 Lyon, France; jacques.portoukalian@univ-lyon1.fr

**Keywords:** linoleic acid, stratum corneum, dogs, ichthyosis, hydroperoxide

## Abstract

Golden Retrievers may suffer from *Pnpl1*-related inherited ichthyosis. Our study shows that in the stratum corneum (SC) of ichthyotic dogs, linoleic acid (LA) is also present in the form of 9-keto-octadecadienoic acid (9-KODE) instead of the acylacid form as in normal dogs. The fatty acids purified from SC strips (LA, acylacids) were characterized by liquid chromatography-tandem mass spectrometry (LC-MS) and atmospheric pressure chemical ionization (APCI). Electrospray ionization (ESI) and MS2(MS/MS Tandem mass spectrum/spectra)/M3 (MS/MS/MS Tandem mass spectrum/spectra) fragmentation indicated the positions of the double bonds in 9-KODE. We showed that ichthyotic dogs have a threefold lower LA content in the form of acylacids. The MS2 fragmentation of acyl acids showed in some peaks the presenceof an ion at the *m*/*z* 279, instead of an ion at *m*/*z* 293 which is characteristic of LA. The detected variant was identified upon MS3 fragmentation as 9-keto-octadecadienoic acid (9-KODE), and the level of this keto-derivative was increased in ichthyotic dogs. We showed by the APCI that such keto forms of LA are produced from hydroperoxy-octadecadienoic acids (HpODE) upon dehydration. In conclusion, the free form of 9-KODE was detected in ichthyotic SC up to fivefold as compared to unaffected dogs, and analyses by HPLC (High performance liquid chromatography) and ESI-MS (Electrospray Ionization-Mass Spectrometry) indicated its production via dehydration of native 9-HpODE.

## 1. Introduction

Linoleic acid is one of the two essential fatty acids for humans and other mammals, the second being alpha-linolenic acid. These fatty acids are an essential dietary requirement since they cannot be synthesized due to a lack of the desaturase enzymes required for their production. Linoleic acid (LA) C18:2 is necessary for the formation of an efficient cutaneous barrier, and a deficiency in linoleic acid supply from the diet leads to scaly skin and a high transepidermal water loss (TEWL) [1]. As shown in our recent study [2], LA is found in canine SC mainly as an ester to omega-hydroxy very long chain fatty acids (ω-OH-VLCFA) in acylacids [3] that form the fatty acid moieties of acylceramides [4]. The occurrence of the latter compounds in the epidermis is critical to ensure an optimal cutaneous barrier function [5].

Autosomal recessive congenital ichthyosis (ARCI) is a condition seen in Golden Retrievers with no difference between males and females [6]. A frameshift variant is found in the *Pnpl1* gene, encoding [7] a transacylase essential for the biosynthesis of acylceramides [8]. ARCI may also be caused by genetic variants in the CERS3 gene encoding ceramide synthase 3 [9]. Deficiency in these enzymes results in a significant decrease in acylceramides in the SC [10,11]. Since LA is by far the most abundant fatty acid esterified on acylceramides [3], our recent findings showing that the increased LA content in the SC of dogs fed an LA-enriched diet is mostly reflected in acylacids [2] prompted us to analyze the acylacid fraction of the SC of ichthyotic Golden Retrievers. LA was found to be about threefold lower compared to unaffected dogs’ SC. Our results firstly highlight the LA acylacids in ichthyotic Golden Retrievers SC versus unaffected SC dogs. Further, the MS/MS studies show acylsacid fragmentation into LA hydroperoxides. In the last part of the results, we identify the oxidized form of LA versus the standard format *m*/*z* 293 in ichthyotic dogs SC. Moreover, we report here the presence of significant levels of oxidized derivatives of LA in acylacids of ichthyotic dogs, much higher than in control dogs.

## 2. Results

### 2.1. LA Acylacid LC-MS-APCI Identification and MS2 Fragmentation

**Figure 1:**
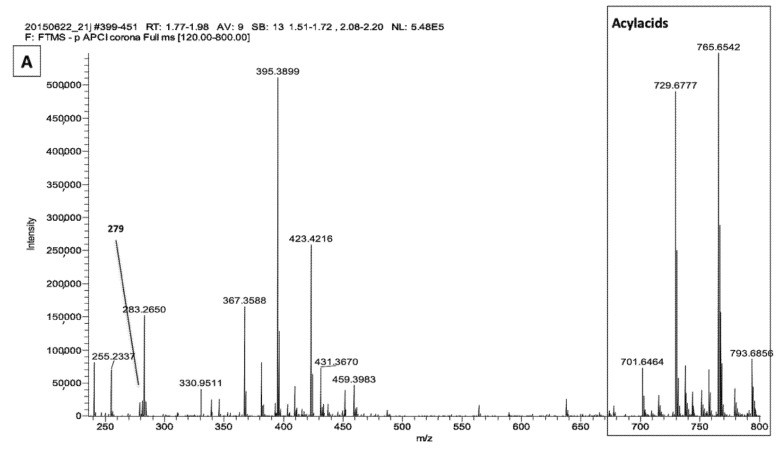

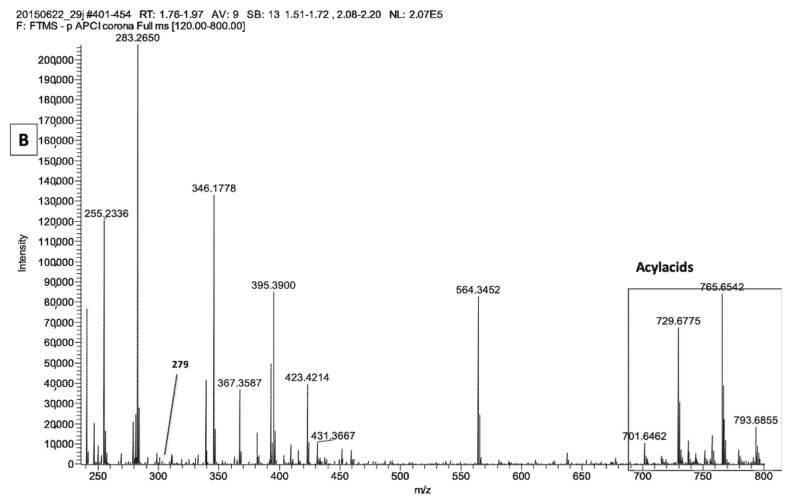
Analysis by APCI-MS of the free fatty acid fraction of canine stratum corneum from *m*/*z* 240 to *m*/*z* 800. Panel (**A**): healthy dog; panel (**B**): ichthyotic dog.

As reported in our recent study [2], the free fatty acid fractions of canine SC analyzed by mass spectrometry with APC (atmospheric-pressure chemical ionization) showed that the major part of LA was esterified to ω-OH-VLCFA in acylacids that produce ions in the *m*/*z* 700 to *m*/*z* 830 range. Figure 1 shows representative spectra obtained with the SC free fatty acid fractions from unaffected (Figure 1A) and ichthyotic dogs (Figure 1B).

The assay of LA gave very low values for the content of ichthyotic dogs SC, in which LA was found to be about threefold lower as compared to healthy dogs (Table 1). Nevertheless, the acylacid form of LA represented the bulk of this fatty acid in ichthyotic as well as in healthy dogs. MS/MS fragmentation enabled the identification of the acylacid ions.

The major ions (M+H) and their Cl adducts arising from the injection in chloroform, as well as their fragments obtained upon MS/MS fragmentation, can be seen in Table 2. Most acylacid ions yielded upon MS2 a fragment at *m*/*z* 279 characteristic of LA (C18:2) and fragments of various ω-OH-VLCFA (from C 28:0 to C34:1), whereas a different result was obtained with two of these ions at *m*/*z* 701.653 and *m*/*z* 743.630, as well as their respective Cl adducts at *m*/*z* 751.637 and *m*/*z* 779.630. Both ions that were present in the SC of healthy and ichthyotic dogs yielded instead an unexpected *m*/*z* 293 ion along with a fragment at *m*/*z* 467.41 corresponding to ω-OH C30:0 fatty acid. Both ions were submitted to further MS3 fragmentation to allow proper identification.

**Figure 2:**
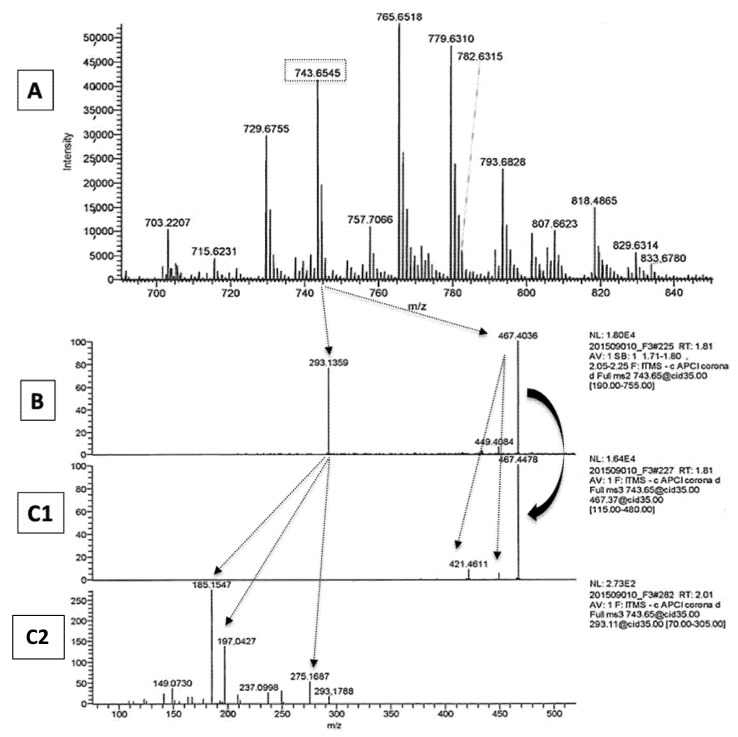
Panel (**A**): Enlarged spectrum of the acylacid fraction of SC from a healthy dog analyzed by APCI-MS; panel (**B**): MS2 fragmentation of the peak at *m*/*z* 743.65 seen on panel A; panel (**C1**): MS3 fragmentation of the peak at 467 (ωOH-C30:0); panel (**C2**): MS3 fragmentation of the peak at *m*/*z* 293.

Figure 2 shows as an example taken from a healthy dog of the major ions of the acylacid fraction of SC (panel A), the MS2 fragmentation of the *m*/*z* 743.65 ion (panel B) that gave two ions at *m*/*z* 467.42 (assigned as ωOHC30:0) and *m*/*z* 293.15, and the MS3 fragmentation of the latter ones that can be seen, respectively, in panel C1 and C2. The MS3 analysis of the ion at *m*/*z* 293.15 gave a spectrum that was almost perfectly superimposable onto the spectrum of 9-keto-octadecadienoic acid (9-KODE) published by Oliw et al. [12]. The MS3 spectrum showed some major signals at *m*/*z* 209 (^-^OOC-(CH_2_)_7_-C(OH)=CH-CH=C), *m*/*z* 197 (^-^OOC-(CH_2_)_7_-CO-CH=CH_2_), and *m*/*z* 185 (^-^OOC-(CH_2_)_7_-C(OH)=CH_2_) that were assigned accordingly [12].

Table 3 shows that the proportions of LA as keto derivatives in the five SC samples of control dogs were in the 3% to 5% range, whereas they increased to the 12% to 16% range in ichthyotic dogs. It means that, although the concentration of LA is lowered in ichthyotic dogs’ SC, one of LA’s keto derivatives is not modified by the disease. In our recent study, the free fatty acid fractions of five control dogs increased their LA content by two-fold after being fed 12 weeks with an LA-enriched diet [2]. Interestingly, despite the significant increase in LA in the SC of those dogs, the proportions of keto derivatives in LA remained in the same 3% to 5% range as the control dogs from this study.

### 2.2. The LA Hydroperoxide Identification

The formation of oxygenated derivatives from esterified LA in the epidermis depends mostly on the activity of the two lipoxygenases, 12R-lipoxygenase (12R-LOX) and epidermal lipoxygenase 3 (eLOX3) [13]. LA, which must be in an esterified form to be a substrate [14], is oxidized by 12R-LOX to give the specific hydroperoxide. The latter is converted by eLOX3 into a specific epoxyalcohol and a keto product. In the present study using mass spectrometry, the MS2 fragmentation of the putative keto derivative at *m*/*z* 293 did not show major ions at *m*/*z* 171 and *m*/*z* 211 that could suggest the presence of epoxyalcohols derived from LA, as outlined by Oliw et al. [12]. Therefore, the action of eLOX3 to account for the detection of an MS2 fragment at *m*/*z* 293 was unlikely.

**Figure 3:**
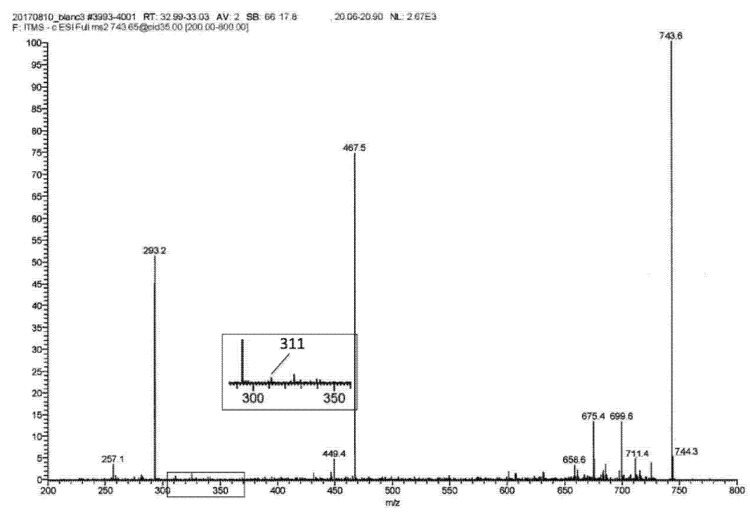
Analysis by ESI-MS of the acylacid fraction of SC from the control dog is shown in Figure 2. MS2 fragmentation of the same peak at *m*/*z* 743.65 is seen in Figure 2. Insert: enlarged spectrum from *m*/*z* 285 to *m*/*z* 360. The peak at *m*/*z* 311 corresponds to LA hydroperoxide.

As an alternative explanation, the experimental conditions of mass spectrometry with APCI are known to induce a loss of H_2_O from hydroperoxides, giving rise to keto derivatives [12]. Therefore, additional experiments were performed with electrospray ionization (ESI) that uses much milder experimental conditions to analyze the unstable hydroperoxide moieties [15]. The resulting MS2 spectrum (obtained by APCI-MS) of the acylacid ion at *m*/*z* 743.6 presented in Figure 2B, and performed again by ESI-MS, can be seen in Figure 3. A small fragment at *m*/*z* 311 can be seen, alongside the major one at *m*/*z* 293, suggesting the presence of linoleic acid hydroperoxides remaining in the experimental conditions of ESI, while this fragment was undetectable using APCI conditions (Figure 1, panel B).

**Figure 4:**
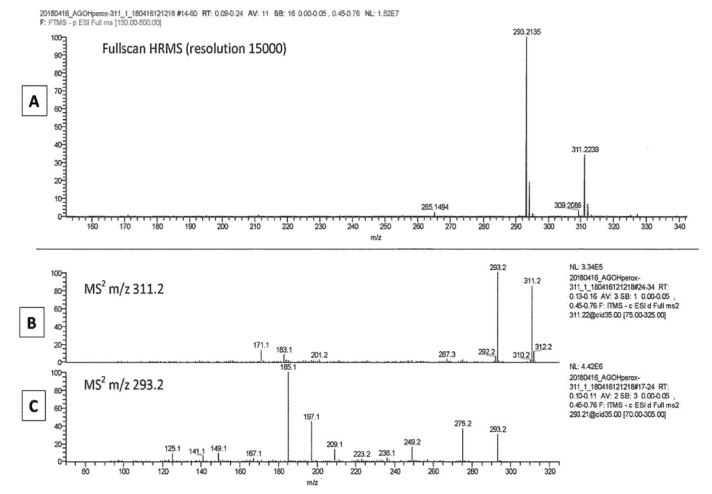
Analysis by ESI-MS of a standard of 9-HpoDE. Panel (**A**): full-scan MS; panel (**B**): MS2 fragmentation of the peak at *m*/*z* 311.2 seen in panel (**A**); panel (**C**): MS2 fragmentation of the peak at *m*/*z* 293.2 seen in panel (**A**).

When the same fragmentation with ESI was applied to an authentic standard of 9(S)-HpODE, the spectra presented in Figure 4 (Panel A) show that a significant part of the native hydroperoxide (at *m*/*z* 311) lost a molecule of water to give the ion at *m*/*z* 293 that, after MS2 fragmentation, yielded a spectrum characteristic of 9-KODE (see Figure 1, Panel C) with major signals at *m*/*z* 185, *m*/*z* 197 and *m*/*z* 249 (Figure 4C). It was noticeable that MS2 of the remaining standard at *m*/*z* 311 (Figure 4B) yielded two small peaks at *m*/*z* 171 and *m*/*z* 201 that are characteristic of epoxides, according to Oliw et al. [12].

**Figure 5:**
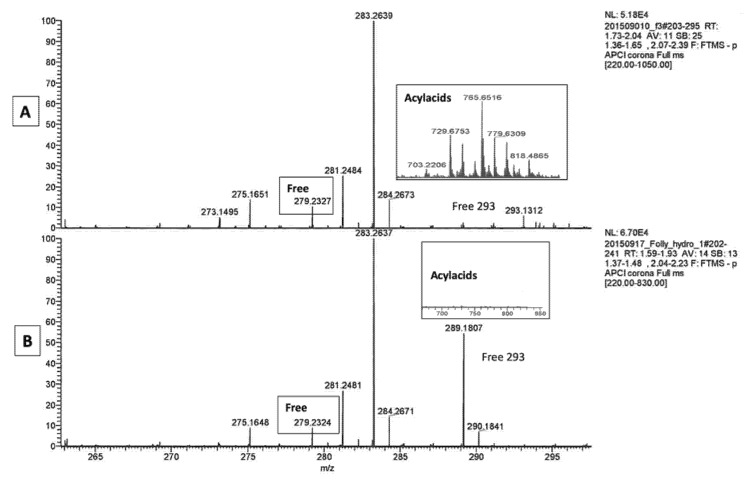
Panel (**A**): enlarged spectrum of the free fatty acid fraction of SC of an ichthyotic dog analyzed by APCI-MS, from *m*/*z* 265 to *m*/*z* 295; Panel (**B**): spectrum of the same sample following saponification, showing the absence of the peak at *m*/*z* 293 seen in panel (**A**), and no change in the peak of C18:2 at *m*/*z* 279 despite the disappearance of the acylacids (shown in inserts) upon saponification.

An additional point of interest in the free fatty acid fraction of SC from ichthyotic Golden Retrievers was the detection by mass spectrometry of a peak of free fatty acid at *m*/*z* 293 (Figure 5A). Such a peak was undetectable in normal dogs. Figure 5B shows that this peak disappeared upon a saponification event that cleaved all remaining acylacids (see inserts in Figure 5A,B) without any change in the size of the LA peak at *m*/*z* 279, thus demonstrating that the chemical hydrolysis of LA esterified in acylacids does not allow recognition of structures, in accordance with the observations of Butovich et al. [16].

**Figure 6:**
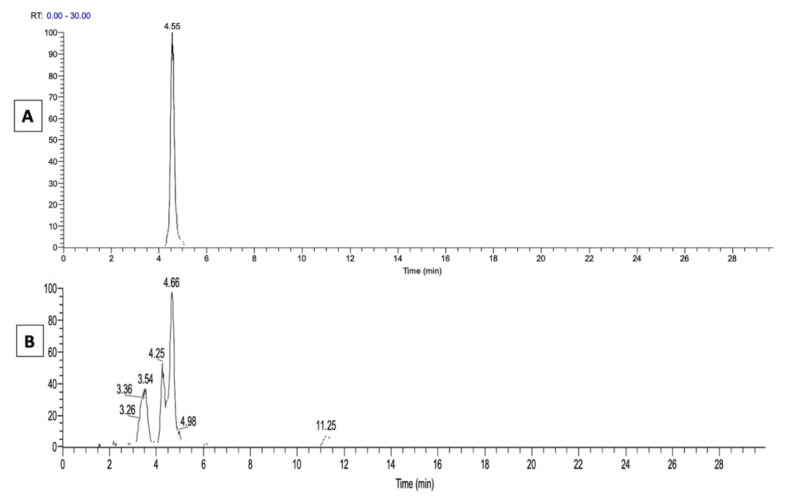
Retention times (EIC) of the ion at *m*/*z* 293.21 detected by ESI-MS in negative mode. Panel (**A**): 9-HpODE standard; Panel (**B**): free fatty acid fraction of an ichthyotic dog SC.

Additional LC-MS experiments were carried out to find out whether the native 9-HpODE is present in the fatty acids of ichthyotic SC. Samples of ichthyotic SC fatty acids were run along with standard 9-HpODE on HPLC and analyzed by MS. As 9-HpODE in SC fatty acids was not visible on the total ion current (TIC) chromatogram (not shown), the extracted ion chromatogram (EIC) of the ion at *m*/*z* 293.21, which is produced by the loss of one hydrogen from the ion at *m*/*z* 311.22 of 9-HpODE (MW = 312), was selected to determine its retention time. Figure 6 shows the retention time of the LA hydroperoxide standard (Panel A in Figure 6) compared to those of the ion at *m*/*z* 293.21 found in ichthyotic dogs SC (Panel B in Figure 6).

**Figure 7:**
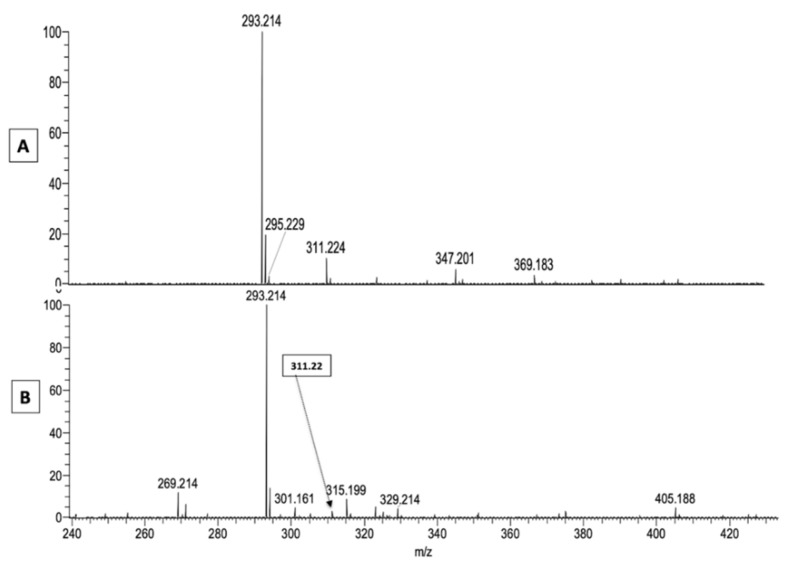
Spectra obtained by ESI-MS for the peaks at 4.6 min retention time (Figure 6). Panel (**A**): 9-HpODE standard; panel (**B**): free fatty acid fraction of an ichthyotic SC.

**Figure 8:**
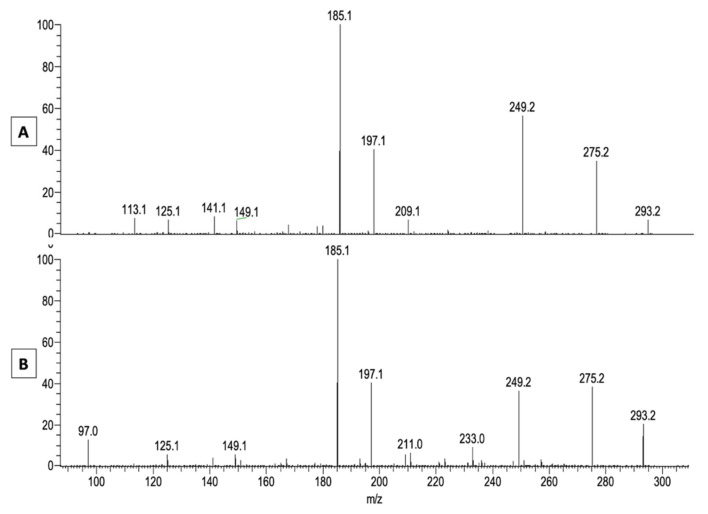
ESI-MS2 fragmentation of the peaks at *m*/*z* 293.214 seen in Figure 7. Panel (**A**): 9-HpODE standard; Panel (**B**): free fatty acid fraction of an ichthyotic SC.

The latter ion is visible at several retention times, suggesting the presence of differently oxidized LA in the sample. Nevertheless, a major peak can be seen with the same retention time at 4.6 min as the 9-HpODE standard. The spectra obtained by ESI-MS for the same peaks with retention time at 4.6 min are presented, respectively, in panels A and B in Figure 7. The ion at *m*/*z* 293.21 is a major one in both standard and canine samples, and the ions of the native products at *m*/*z* 311.22 are visible in the standard and also in the ichthyotic dog SC, albeit in much smaller amounts. The peaks at *m*/*z* 293.21 of both samples seen in Figure 7 were submitted to ESI-MS2 fragmentation, and they yielded spectra (Panels A and B in Figure 8) that are similar to the one shown in Figure 4C, with some additional peaks at *m*/*z* 211 and *m*/*z* 233 in the ichthyotic sample.

These data suggest that the free fatty acid fraction of ichthyotic dogs indeed contains some 9-HpODE that is dehydrated to give the ion at *m*/*z* 293. It is, therefore, likely that the ion at *m*/*z* 293 found by mass spectrometric fragmentation of the acylacids of normal and ichthyotic dogs’ stratum corneum results from the decomposition of 9-hydroperoxyoctadecadienoic acid esterified on ω-OH-VLCFA.

## 3. Discussion

Our results show that the LA content of canine SC is much lower in ichthyotic Golden Retriever dogs as compared to unaffected control dogs. Sex had no influence on the results, which were in the same range for male and female dogs. It was previously observed in a recent study that there are no significant sex- or breed-related differences in Stratum Corneum ceramides [17,18]. It has been reported recently that the epidermal LA content of *Pnpla1*^-/-^ mice does not change with their *Pnpla1* genotype [10], but this discrepancy can be explained by the fact that the whole epidermis was analyzed, instead of the SC as in our study, and only the free LA was assayed by LC-MS, while LA in acylacids was not taken into account. Our finding regarding the very low acylacid content of the SC of ichthyotic Golden Retrievers is consistent with the results of Hirabayashi et al. obtained with the whole epidermis of *Pnpla1* deficient mice [11]. The *Pnpla1* gene variant affects the activity of the transacylase that, in healthy conditions, esterifies LA on ω-hydroxy-glucosylceramides in the stratum granulosum [8,10,11]. The acylglucosylceramides are then cleaved into acylceramides that are the major source of LA in the SC of healthy dogs. In ichthyotic Golden Retriever dogs, the content of acylceramide is very low [8,10] and LA-containing acylacids cannot be produced upon cleavage of the sphingoid base. As an additional finding to emphasize the importance of LA esterified on acylacids and acylceramides, it was shown by Ohno et al. [8] that an exogenous supply of free LA does not improve the acylceramide synthesis in *Pnpla1*^-/-^ transfected HEK293T cells. It is a feature that is consistent with the fact that the LA esterified to ω-OH-VLCFA in acylceramides must be oxidized by lipoxygenases to allow hydrolysis of the esterified LA and bonding of the ω-OH-ceramides to proteins [19], taking into account that free LA is not a substrate for these enzymes that oxidize only esterified LA [13].

Although little is known about the lipoxygenases in canine skin, the lipoxygenase 12R-LOX converts the esterified linoleic acid to the hydroperoxy derivative, then the action of ELOX3 leads to the formation of epoxyalcohols and keto-esters of the ceramide [14]. However, these oxygenated derivatives have thus far been described as part of ceramides, although Zheng et al. reported the presence of epoxy-keto ω-OH-VLCFA in pig epidermis following the extensive overnight extraction of lipids [17]. The present study shows the presence of oxygenated derivatives of LA that is esterified on ω-OH-VLCFA in canine SC. The results suggest that the major oxygenated derivative is actually the 9-hydroperoxy-octadecadienoic acid that is dehydrated to the 9-keto-derivative during the analyses by mass spectrometry. However, the absence of epoxyalcohols in the SC of unaffected and ichthyotic Golden Retriever dogs rules out any effect of eLOX3 on the esterified hydroperoxy-octadecadienoic acid (HpODE), and since the lipoxygenases 12R-LOX and eLOX3 act sequentially [18,19], these enzymes are unlikely to be the source of the oxidized derivatives of LA found in our study. In this hypothesis, autoxidation would be the only alternative explanation for our finding, as hydroxyoctadecenoic acid has been reported to be a biomarker of lipid peroxidation in vivo [20]. The presence of an oxidized form of LA as a free acid at *m*/*z* 293 in icthyotic dogs’ SC may also be due to autoxidation since non-esterified LA is not a substrate for lipoxygenases [15]. The fact that the proportion of oxidized derivatives of LA is much higher in the SC of ichthyotic Golden Retrievers could result from cutaneous inflammation since such a skin condition is known to induce the production of oxidized derivatives of LA [21]. Oxidized LA metabolites have been reported to activate transient receptor potential cation channel subfamily V member 1 (TRPV1) [22], which are receptors known to be abundant in epidermal keratinocytes and skin nerve endings [23], and TRPV1 activation delays cutaneous barrier recovery [24]. As to the fate of oxidized derivatives of LA esterified to ω-OH-VLCFA, the possibility of a transesterification on proteins remains questionable. The analysis of lipopeptides purified from human foreskin epidermis showed that they were made of ceramides coupled to involucrin and some other proteins [25], but the authors did not report ω-OH-VLCFA as parts of the lipopeptides, despite the fact that these fatty acids account for a significant amount (about half the content in ω-OH-ceramides) of the protein-bound lipids released in alkaline conditions from human epidermis [26].

In any case, the presence of oxygenated derivatives of LA in acylacids of SC is an interesting observation that deserves additional studies to obtain new insight into the biological activity of these derivatives.

## 4. Materials and Methods

### 4.1. Tissues

Samples of SC were obtained at VetAgro Sup (Marcy L’Etoile, France) by tape stripping (10 tapes each) applied to the abdomens of 5 healthy adult male Golden Retrievers and 5 ichthyotic male Golden Retrievers, as previously described [2]. The strips from the SC were taken during a therapeutical intervention at the Veterinary School.

### 4.2. Lipid Extraction and Analysis

In order to get rid of the adhesive with a minimal loss of cellular lipids, each tape was dipped in a glass tube with 10 mL of hexane-isopropanol. After sonication for 5 min in a Bransonic 2000 bath sonicator (Branson, Paris, France), the tapes were discarded, and the released corneocytes were pelleted by centrifugation 10 min at 1500× *g*. The corneocytes from the tapes of each individual were pooled, and the lipids were extracted for 1 h in methanol-chloroform 2:1 (*v*/*v*). After centrifugation, the supernatants were collected. The protein pellets were extracted with methanol only twice more for 2h each time. The supernatants were pooled and evaporated under nitrogen in a Rotavapor (Büchi, Flawil, Switzerland) at 37 °C. The lipids were taken up in 0.5 mL of diethylether and applied onto LC-NH2 silica gel cartridges (Supelco, L’Isle d’Abeau, France) that were then eluted as described [2]. Briefly, neutral lipids, ceramides, free fatty acids, and anionic lipids were sequentially eluted with, respectively, chloroform, chloroform-methanol 23:1 (*v*/*v*), diisopropylether-acetic acid 98:3 (*v*/*v*), and 0.2% ammonium acetate in methanol. The purity of the free fatty acid fractions was checked by thin-layer chromatography on silica gel thin-layer plates (Merck, Darmstadt, Germany) developed in chloroform-methanol-acetic acid 95:5.5:0.5 (by volume). The lipids were visualized with a spray reagent (3% copper acetate in 8% phosphorous acid) by heating in an oven at 150 °C.

### 4.3. Analysis of Free Fatty Acids by Liquid Chromatography-Mass Spectrometry

The fatty acid fractions extracted from SC tape strips and purified by chromatography on the LC-NH2 cartridges were taken up in chloroform and analyzed by liquid chromatography-mass spectrometry (LC-MS) without derivatization as described [2] after the addition of 2 µg of decapentanoic acid (C15:0 as a standard for quantification). A 5 µL sample was injected in an RSLC Dionex-U300 column coupled to an LTQ-Orbitrap Velos Pro mass spectrometer (Thermofisher Scientific, Villebon-sur-Yvette, France) equipped with an atmospheric-pressure chemical ionization (APCI) interface. The mass spectra were recorded with high resolution (100,000) from *m*/*z* 200 to *m*/*z* 800. For mass spectrometry using electrospray ionization (ESI) in negative mode, the samples were taken up in methanol-water 9:1 (*v*/*v*) and injected in an Acclaim Dionex RSLC 120-C18, 2.2 µm, 120A, 2.1 × 150 mm. Standard 9(S)-hydroperoxy-octadecadienoic acid (9S-HpODE) was purchased from Cayman Chemical (Ann Arbor, MI, USA) and used as a control.

## 5. Conclusions

The present study shows the presence of oxygenated derivatives of LA esterified onto ω-OH-VLCFA in canine SC, mostly in the 9-hydroperoxy-octadecadienoic acid (9-KODE) form, which is dehydrated to the 9-keto-derivative during the analyses by mass spectrometry. The 9-KODE was increased up to five-fold in the SC of *Pnpla1*-deficient ichthyotic dogs compared to controls. The absence of epoxyalcohols in the SC of non-affected and ichthyotic dogs rules out any effect of eLOX3 on the esterified hydroperoxy-octadecadienoic acid (HpODE). Since lipoxygenases 12R-LOX and eLOX3 act sequentially, the only alternative explanation for oxidized derivatives of LA *m*/*z* 293 is due to autoxidation triggering a cutaneous inflammation [21].

As hydroperoxy-octadecadienoic acids (9-HpODE) production in SC induces dehydration and cutaneous inflammation, further studies dealing with the metabolic regulation of the substrate or pharmacological targeting of 12R-LOX and eLOX3 could lead to clinical trials aimed at the induction of cutaneous barrier recovery. As previously shown [2], LA can only be stored in the SC in the form of acylacids, and it would be of interest to carry out clinical trials using purified acylacids either in oral treatment or in topical applications on ichthyotic dogs. Moreover, the groups of Leoón-Loópez [27], and Aguirre-Cruz [28], demonstrated that a topical administration with low molecular hydrolyzed collagen (HC) from sheepskins is a natural antioxidant that showed good antioxidant activity to neutralize the deleterious effect of oxidation by preventing the propagation step of oxidation. This finding could be useful to investigate the putative mechanism of autoxidation of LA derivatives in the skin.

## Figures and Tables

**Table 1:** LA content in canine stratum corneum of dogs. Percentage as free fatty acids and acylacids as recorded from *m*/*z* 700 to *m*/*z* 900 MS spectra. The corresponding percentages are shown after APCI-LC-MS comparison data.

Dogs (*n* = 5)	C18:2 (µg/mg Proteins)	% as Free Fatty Acid	% as Acylacid
Control dogs	26.7 ± 14.3	8.6 ± 6.4	91.3 ± 12.6
Ichthyotic dogs	8.1 ± 5.6	10.2 ± 7.5	90.1 ± 13.8

**Table 2:** Results of the MS2 fragmentation of acylacids recorded from *m*/*z* 700 to *m*/*z* 900. The corresponding fragments, formulas and the molecular weights (M-H and M+Cl) are shown after APCI-LC-MS and -MS2.

M-H	M+Cl	Formula	Fragments	Structures
701.643	737.620	C46H85O4	439; 421; 279	(O-C18:2)-ωOH-C28:0
715.659	751.659	C47H87O4	439; 421; 293	(O-ketoC18:2)-ωOH-C28:0
729.674	765.651	C48H89O4	467; 449; 279	(O-C18:2)-ωOH-C30:0
743.637	779.630	C48H88O5Cl	467; 293	(O-ketoC18:2)-)-ωOH-C30:0
743.693	779.666	C49H92O4Cl	481; 463; 279	(O-C18:2)-ωOH-C31:0
783.700	819.700	C52H96O4Cl	521; 503; 279	(O-C18:2)-ωOH-C34:1

**Table 3:** Percentages of LA found as keto-derivatives in canine stratum corneum.

Dogs (*n* = 5)	% LA as Keto-Derivatives
Control dogs	3.7 ± 0.6
Control dogs fed with LA-enriched diet [2]	3.8 ± 0.7
Ichthyotic Golden Retrievers dogs	13.9 ± 1.8

## Data Availability

The data presented in this study are available in insert article.
